# Die HODOKORT-Studie und aktuelle Aspekte der Hörsturztherapie mit Glukokortikoiden

**DOI:** 10.1007/s00106-024-01458-3

**Published:** 2024-04-10

**Authors:** Stefan K. Plontke

**Affiliations:** Universitätsklinik und Poliklinik für Hals-Nasen-Ohren-Heilkunde, Kopf- und Hals-Chirurgie, Martin-Luther-Universität Halle-Wittenberg, Ernst-Grube-Str. 40, 06120 Halle (Saale), Deutschland

Mit dem Abschluss der HODOKORT-Studie (Effektivität und Sicherheit der systemischen HOchDOsis-GlukoKORTikoidtherapie beim Hörsturz – eine dreiarmige, multizentrische, randomisierte, tripelblinde Studie; https://hodokort-studie.hno.org) und dem Cochrane-Review zur intratympanalen Therapie des Hörsturzes mit Glukokortikoiden ergeben sich im Vergleich zur früheren Leitlinie „Hörsturz“ der Arbeitsgemeinschaft medizinisch-wissenschaftlicher Dachgesellschaften (AWMF) neue Aspekte in der Therapie des akuten idiopathischen sensorineuralen Hörverlusts („Hörsturz“) [1–4]. Hier sollen einige dieser Aspekte aufgegriffen, in Kürze kurz zusammengefasst und Hinweise für sich aus den neuen Erkenntnissen ergebende, mögliche Vorgehensweisen dargestellt werden. Die in Bearbeitung befindliche AWMF-Leitlinie soll dann detaillierte und konsentierte Empfehlungen geben.

## HODOKORT-Studie zur systemischen Hochdosis-Glukokortikoid-Therapie

Im Rahmen der vom Bundesministeriums für Bildung und Forschung (BMBF) im „Rahmenprogramm klinische Studien mit besonderer Relevanz für die Patientenversorgung“ geförderten HODOKORT-Studie wurden Patienten mit Hörsturz von 50 dB oder mehr in den drei am meisten betroffenen benachbarten Frequenzen innerhalb von 7 Tagen nach Beginn des Hörsturzes entweder einer jeweils 5‑tägigen Therapie mit hochdosiertem Prednisolon (250 mg/Tag intravenös, HD-Pred), hochdosiertem Dexamethason (40 mg/Tag oral, HD-Dex) oder niedriger dosiertem Prednisolon (60 mg/Tag oral, Pred-Control), Letztere gefolgt von einer 5‑tägigen Dosisreduktion, zugewiesen. Der primäre Zielparameter war die Veränderung der Hörschwelle gemessen als Mittelwert der drei am stärksten betroffenen benachbarten Frequenzen (3PTA_meist betroffen_) vom Ausgangswert bis zum Tag 30. Zu den sekundären Zielparametern gehörten Sprachverständnis, Tinnitus, Kommunikationskompetenz, Lebensqualität, Bluthochdruck und Insulinresistenz [3].

Insgesamt wurden 325 Patienten randomisiert. Von den 325 randomisierten Patienten mit Hörsturz hatten 17 (5 %) ein Vestibularisschwannom als Ursache des plötzlichen Hörverlusts. Diese wurden nicht mit in die Auswertung der primären und sekundären Zielparameter einbezogen (modifizierte Intention-to-Treat[ITT]-Population: *n* = 312).

Die durchschnittliche Veränderung der Hörschwelle (3PTA_meist betroffen_) betrug 34,2 dB (95%-Konfidenzintervall, 95%-KI: 28,4 bis 40,0) in der der HD-Pred-Gruppe, 41,4 dB (95%-KI: 35,6–47,2) in der HD-Dex-Gruppe und 41,0 dB (95%-KI: 35,2–46,8) in der Pred-Kontrollgruppe (*p* = 0,09 für die Varianzanalyse). Der Unterschied in der Veränderung betrug −6,8 dB (95%-KI: −15,1 bis 1,4) zwischen HD-Pred und Pred-Control, 0,5 dB (95%-KI: −7,8 bis 8,7) zwischen HD-Dex und Pred-Control und −7,2 dB (95%-KI: −15,5 bis 1,0) zwischen HD-Pred und HD-Dex.

Die Teilnehmer der HD-Pred-Gruppe wiesen zu jedem Zeitpunkt schlechtere absolute Hörschwellen und geringere Verbesserungen auf als die Teilnehmer der HD-Dex- und der Pred-Kontrollgruppe (3PTA_meist betroffen_, 3PTA_0,5–2_ _kHz_ und 4PTA_0,5–4_ _kHz_). In der HD-Pred-Gruppe hatten weniger Teilnehmer (24 %) am Tag 30 eine vollständige Hörverbesserung als in der HD-Dex- (38 %) oder Pred-Kontrollgruppe (39 %). Die HD-Pred- und HD-Dex-Gruppen wiesen 30 Tage nach Beginn der Therapie ein schlechteres Sprachverständnis im Freiburger Einsilbertest in Ruhe auf (HD-Pred 57,4 ± 41,4 %; HD-Dex 61,1 ± 41,3 %) als die Kontrollgruppe mit niedriger Dosierung (70,4 ± 39,6 %).

In den Gruppen HD-Pred (*n* = 73) und HD-Dex (*n* = 76) traten mehr unerwünschte Ereignisse im Zusammenhang mit der Studienmedikation auf als in der Pred-Kontrollgruppe (*n* = 46).

Ein Auszug der Ergebnisse findet sich in Tab. 1. Die ausführlichen Ergebnisse sind in der Hauptpublikation für die HODOKORT-Studie und dem dazugehörigen Appendix dargestellt [1].

**Table 1:** 

	Hochdosis-Prednisolon (i.v.)	Hochdosis-Dexamethason (p.o.)	Kontrolle Prednisolon (p.o.)	
**Ausgangswerte**				**Gesamt**
Anzahl der Patienten (modifizierte ITT-Population)^a^	101	105	102	308
Alter, MW (Std.) in Jahren	57,3 (± 13,4)	54,1 (± 15,1)	55,1 (± 13,7)	55,5 (± 14,1)
Hörverlust (3 meisten betroffene Frequenzen); MW (Std.)	80,1 (± 20,4) dB HL	81,1 (± 21,3) dB HL	77,7 (± 19,4) dB HL	79,7 (± 20,4) dB HL
ES@65 dB MW (Std.)	18,6 (± 28,4) %	15,5 (± 27,9) %	17,8 (± 28,1) %	17,3 (± 28,0) %
**Primärer Zielparameter**				***p***^**§**^
Änderung der Hörschwelle^b^	34,2 (± 25,8) dB	41,4 (± 26,2) dB	41,0 (± 26,0) dB	0,09
**Sekundäre Zielparameter** (Auswahl)				
Endwert Hörschwelle^b^ (6 Monate)	40,7 (± 26,4) dB HL	34,2 (± 26,1) dB HL	31,3 (± 26,1) dB HL	0,07
Endwert ES@65 dB MW (Std.) (6 Monate)	61,0 (41,0) %	68,7 (39,7) %	74,4 (36,4) %	0,05
Anteil mit vollständiger Erholung	24 %	38 %	39 %	–
Änderung Tinnitus-Belästigung^c^ (6 Monate), Median [25.; 75. Perzentile]	−10,0 [−32,0; −1,0]	−30,0 [−57,0; −7,0]	−30,0 [−46,0; −5,0]	0,05
Änderung Tinnitus-Lautstärke^c^ (6 Monate), Median [25.; 75. Perzentile]	−10,0 [−25,5; 1,0]	−23,0 [−44,0; 0,0]	−25,0 [−40,0; −10,0]	0,01

*ES* Verständlichkeit in % im Freiburger Einsilbertest bei 65 dB SPL (Sound Pressure Level), *MW* Mittelwert, *Std.* Standardabweichung

^a^ITT: Intention-to-Treat-; modifiziert, da 17 Teilnehmer (5 %) mit Vestibularisschwannom von 325 randomisierten ausgeschlossen

^b^Bezogen auf die 3 am meisten betroffenen Frequenzen, positive Werte bedeuten eine Verbesserung

^c^Tinnitus: negative Werte bedeuten eine Verbesserung

^§^**Cave** (*p*-Werte) Studie nicht ausgelegt (nicht „gepowert“) für sekundäre Zielparameter

## Cochrane-Review zur intratympanalen Therapie des Hörsturzes mit Glukokortikoiden

Ebenfalls mit Förderung des BMBF erfolgte im Rahmen des Forschungsprojekts ITKORT ein „systematic review“, d. h. eine systematische Auswertung und Metaanalyse bereits durchgeführter, randomisierter klinischer Studien zur Hörsturzbehandlung mittels intratympanaler Glukokortikoidapplikation [4].

Im besonderen Fokus standen folgende Fragen: Hat die intratympanale Therapie – die Injektion von Medikamenten hinter das Trommelfell, direkt an die Gehörschnecke – Vorteile gegenüber einer systemischen Medikamentengabe, in der Regel in Tablettenform? Welche Behandlungsform ist zur Erstbehandlung (Primärtherapie) am besten geeignet und bringen intratympanale Injektionen eine Verbesserung, wenn die Erstbehandlung nicht erfolgreich war (Sekundärtherapie)? Und schließlich: Kann eine Kombination aus beiden Behandlungsformen den Betroffenen spürbar, d. h. klinisch relevant, helfen?

Als Ergebnisse des Cochrane-Reviews ergaben sich die im Folgenden dargestellten Erkenntnisse:

### Primärtherapie eines Hörsturzes

Bei der Primärtherapie eines Hörsturzes führen intratympanale Glukokortikoide im Vergleich zu Personen, die systemische Glukokortikoide erhalten, zu einer zusätzlichen Verbesserung von im Mittel 6 dB, und die endgültige Hörschwelle ist um zusätzlich 3 dB besser im Frequenzbereich, der international üblicherweise zur Beurteilung des Hörverlusts herangezogen wird. Die Wahrscheinlichkeit, sich um mindestens 10 bis 15 dB zu verbessern, hat bei der intratympanalen Therapie nur einen Faktor von 1,04 (4 % höhere Wahrscheinlichkeit). Dies konnte mit geringer bis moderater Sicherheit gezeigt werden. Als minimal wichtiger Unterschied zwischen den Gruppen (MID), d. h. als kleinste Änderung eines Behandlungsergebnisses, die ein einzelner Patient als wichtig erachten würde und die eine Änderung der Behandlung des Patienten anzeigen würde, wurde von den Autoren des Cochrane-Reviews 10 dB (Hörschwellen) bzw. 25 % (RR 1,25 für Wahrscheinlichkeit der Hörverbesserung) festgelegt (zur Problematik des MID s. unten). Die Ergebnisse deuten also darauf hin, dass es keinen zusätzlichen Nutzen alleiniger intratympanaler Glukokortikoide gegenüber systemischer Glukokortikoide bei der Primärtherapie gibt.

Die *Kombinationstherapie (intratympanal plus systemisch)* führte zu Verbesserungen der Hörschwelle und einer verbesserten Endhörschwelle von je zusätzlich 9 dB, und die Wahrscheinlichkeit für eine Hörverbesserung war 1,27-mal so hoch (27 % höhere Wahrscheinlichkeit) wie bei alleiniger systemischer Therapie. Bei diesen Analysen (s. Analysen 2.1 bis 2.3. auf den Seiten 115 und 116 im Cochrane-Review) schlossen die Konfidenzintervalle die Null (Hörschwellenunterschiede) bzw. die 1 (Wahrscheinlichkeit der Hörverbesserung) nicht mit ein. Diese Ergebnisse konnten nur mit geringer Sicherheit gezeigt werden. Die Ergebnisses des Cochrane-Reviews deuten also in Richtung entweder auf keinen oder einen geringen zusätzlichen Nutzen (nur < 10 dB oder 27 %) einer kombinierten Therapie gegenüber systemischen Kortikosteroiden hin. Für diese Aussage liegt allerdings keine hohe Evidenz vor.

### Sekundärtherapie des Hörsturzes

Bei der Sekundärtherapie des Hörsturzes (wenn die erste Behandlung nicht erfolgreich war) führen intratympanale Glukokortikoide im Vergleich zu keiner oder einer Placebobehandlung bei einer viel größeren Anzahl von Personen zu einer Verbesserung des Hörvermögens (mehr als 5‑mal so hohe Wahrscheinlichkeit). Die zusätzliche Verbesserung im Vergleich zu Placebo oder zum „Nichtstun“ betrug 9 dB, und die endgültige Hörschwelle war um 11 dB besser. Bei diesen Analysen (s. Analysen 3.1 bis 3.3. auf den Seiten 118 und 119 im Cochrane-Review) schlossen die Konfidenzintervalle die Null (Hörschwellenunterschiede) bzw. die 1 (Wahrscheinlichkeit der Hörverbesserung) nicht mit ein. Auch wenn Cochrane-Reviews nicht mit Aussagen zur statistischen Signifikanz arbeiten, sondern mit der GRADE-Klassifizierung, sind Rückschlüsse auf die statistische Signifikanz mithilfe des Konfidenzintervalls möglich. Enthält ein Vertrauensbereich den Wert des „Null-Effekts“ nicht, so kann man von einem „statistisch signifikanten“ Ergebnis ausgehen.

Die Ergebnisse des Cochrane-Reviews deuten also auf einen Nutzen einer intratympanalen Sekundärtherapie gegenüber keiner oder einer Placebotherapie hin, auch wenn aufgrund von Qualitätsmängeln der meisten betrachteten Studien bei diesem Vergleich die Sicherheit der Evidenz dafür gering ist. Weitere Studien können diese Erkenntnis in die eine oder andere (entgegengesetzte) Richtung verschieben.

Die Aussage „geringe (quantitative) Verbesserung“ (im Mittel 9–11 dB) im Cochrane-Review bezieht sich auf den für die Cochrane-Analyse notwendigerweise festzulegenden (und vom Autorenteam mit diesem Betrag mit 10 dB selbst auferlegten) „minimalen bedeutsamen Unterschied“ („minimal important difference“, MID) zwischen zwei Gruppen, dessen nicht zu unterschätzende Problematik im Cochrane-Review auf den Seiten 36 bis 37 ausführlich diskutiert wird.

Der gesamte Cochrane-Review ist öffentlich zugänglich:

https://www.cochranelibrary.com/cdsr/doi/10.1002/14651858.CD008080.pub2/full

Da die 139 Druckseiten sicher eine mühsame und zeitaufwendige Lektüre darstellen können, sollen dem interessierten Leser folgenden Abschnitte des Cochrane-Reviews als Auszug dienen, um sich weitgehend mit den wichtigsten Ergebnissen vertraut machen zu können: „Abstract“ (S. 1–3), „Summary of Findings Tables“ (S. 5–13), „Discussion“ (insbesondere S. 36–37), „Forest Plot Analysis“ 1.1–3.3 (S. 108–119), (Abschnitte teilweise zitiert aus [5]).

Ein mögliches, gestuftes Vorgehen auf der Basis der vorliegenden Evidenz, wie es in der Klinik des Autors derzeit in der Regel praktiziert wird, ist in Tab. 2 dargestellt. Eine S2k-Leitlinie zum Hörsturz ist angemeldet und in Bearbeitung.

**Table 2:** 

Hörverlust	Vorgehen
**Gering bis moderat** (z. B. bis 50 dB in den 3 am meisten betroffenen Frequenzen) Siehe Chen et al. 2003^§^	*Oral*e Prednisolon-Therapie (*): z. B. wie bei der HODOKORT-Kontrollgruppe: (5 Tage je 60 mg, dann ausschleichen: 2 Tage je 40 mg und 3 Tage je 20 mg) Alternativ, z. B. bei Kontraindikationen: intratympanale Triamcinolonacetonid-Injektionen (40 mg/ml, 5 Tage je 0,3–0,7 ml täglich; alternativ 5 × in 2 Wo), (**)
**Moderat bis schwer** (ca. ≥ 50 dB in den 3 am meisten betroffenen Frequenzen)	Behandlung möglichst im Rahmen klinischer Studien (***)
**Wenn keine Besserung:** „Salvage-Therapie“ (Daten liegen bis 7 Wochen nach Hörsturz vor)	*Intratympanal*e Triamcinolonacetonid-Injektionen (**) 40 mg/ml, 5 Tage je 0,3–0,7 ml täglich (alternativ 5 × in 2 Wochen) Ggf.* Tympanoskopie* zum Ausschluss Verlegung des runden und/oder ovalen Fensters und Triamcinolonacetonid auf resorbierbarem Schwämmchen (z. B. Curaspon)
**„Kein nutzbares Hörvermögen“** (trotz Hörgerät) **und *wenn nicht im Rahmen klinischer Studien* möglich**	*Kombiniert*e Therapie: Orale Prednisolon-Therapie (s. oben) + intratympanale Triamcinolonacetonid-Injektionen (s. oben) *oder:* + Tympanoskopie (s. unten)
**Nicht mehr messbare Hörschwelle** (± Schwindel)	*Oral*e Prednisolon-Therapie (s. oben) + *Tympanoskopie* und Triamcinolonacetonid auf resorbierbarem Schwämmchen (z. B. Curaspon)

^§^Chen et al. (2003) zeigten, dass aufgrund von „Floor-Effekten“ (Tongehörschwelle) und „Ceiling-Effekten“ (Sprachverstehen) Hörsturzstudien bei geringen Hörverlusten nicht oder kaum möglich sind [11]

*Alternative orale Schemata wären z. B. Prednison 60 mg/Tag für 14 Tage, dann 5 Tage ausschleichen 50 mg, 40 mg, 30 mg, 20 mg, 10 mg [12] oder Prednison, 1 mg/kg und Tag (maximal 60 mg/Tag) *oder* Methylprednisolon 48 mg/Tag *oder* Dexamethason 10 mg/Tag für 7 bis 14 Tage, dann ausschleichen [9]

**Alternative intratympanale Schemata wären z. B. 40 mg/ml Methylprednisolone über 2 Wochen, alle 3 bis 4 Tage eine Injektion [12] oder Dexamethason 24 mg/ml oder 10 mg/ml (wenn 24 mg/ml nicht verfügbar) *oder* Methylprednisolon 40 mg/ml (oder 30 mg/ml): bis zu 4 Injektionen, 0,4 bis 0,8 ml, verteilt über 2 Wochen [9]

***Eine aktuelle internationale und auch in Deutschland laufende Hörsturzstudie ist die AC102-Studie (https://www.audiocure.com/de/klinische-studie-2-2/)

Zu den Problemen der aktuellen Evidenzlage zählt, dass der Nutzen von Glukokortikoiden bei der Therapie des Hörsturzes insgesamt unklar ist [6]. Insbesondere stellt die fehlende Dosis-Wirkungs-Beziehung die Wirksamkeit der Glukokortikoidtherapie auch generell in Frage [1, 7, 8]. Da systemische Glukokortikoide seit fast 50 Jahren als Standardbehandlung bei einem Hörsturz gelten [6, 9, 10], erschien zum Zeitpunkt der HODOKORT-Studie eine Placebogruppe weder ethisch vertretbar noch im Hinblick auf die Erwartungen der Patienten und die Rekrutierung praktisch möglich. Angesichts der Ergebnisse der HODOKORT-Studie ist jedoch eine placebokontrollierte Studie oder – falls dies nicht möglich ist – eine randomisierte, kontrollierte Studie zur Deeskalation der Dosis gerechtfertigt.

Grundsätzlich werden dringend weitere neue medikamentöse Therapiemöglichkeiten bei Hörsturz benötigt. Eine aktuelle internationale und auch in Deutschland laufende Hörsturzstudie ist die AC102-Studie (https://www.audiocure.com/de/klinische-studie-2-2).

## Interessenkonflikt

Die Institution des Autors S.K. Plontke erhält Forschungsunterstützung von Cochlear Ltd. Sydney, Australien, MED-EL, Innsbruck, Österreich, und AudioCure Pharma GmbH, Berlin, Deutschland. Der Autor ist Leiter der klinischen Prüfung bei einer Studie zur Therapie des Hörsturzes (Sponsor: AudioCure Pharma GmbH, Berlin, Deutschland) und zur Cochleaimplantation (Sponsor: MED-EL, Innsbruck, Österreich). Der Autor erhielt Reisekostenunterstützung von Cochlear Ltd. Sydney, Australien, und Honorare für Referententätigkeit von MedKom Akademie GmbH, München; Merck Healthcare Germany GmbH, Weiterstadt, und Biologische Heilmittel Heel GmbH, Baden-Baden (Deutschland), sowie Vortragshonorare von verschiedenen Universitäten und wissenschaftlichen HNO-Fachgesellschaften.
